# Alternative treponemal serology assays for diagnosis and confirmation of syphilis in a diagnostic laboratory: a retrospective evaluation of four agglutination assays and one ELISA

**DOI:** 10.1128/jcm.01768-24

**Published:** 2025-05-09

**Authors:** Eloise Williams, Theo Karapanagiotidis, Suellen Nicholson, Helen Toma, Kim Lynn Vo, Celia Douros, Francesca Azzato, Peta Edler, Maryza Graham, Janet M. Towns, Marcus Y. Chen, Chuan K. Lim, Deborah A. Williamson

**Affiliations:** 1 Victorian Infectious Diseases Reference Laboratory, Royal Melbourne Hospital at the Peter Doherty Institute for Infection and Immunity 90134 , Melbourne, Victoria, Australia; 2 Department of Infectious Diseases, The University of Melbourne at the Peter Doherty Institute for Infection and Immunity 2281 , Melbourne, Victoria, Australia; 3 Melbourne Sexual Health Centre, Alfred Health 5392 , Melbourne, Victoria, Australia; 4 Department of Microbiology, Monash Health 214149 , Clayton, Victoria, Australia; 5 Faculty of Medicine Nursing and Health Sciences, Monash University 22457 , Melbourne, Victoria, Australia; 6 School of Translational Medicine, Monash University 2541 , Melbourne, Victoria, Australia; 7 School of Medicine, University of St Andrews 12187 , St Andrews, Scotland, United Kingdom; Marquette University, Milwaukee, Wisconsin, USA

**Keywords:** syphilis, serology, treponemal, sexually transmitted infection, diagnostics

## Abstract

The *Treponema pallidum* particle agglutination (TPPA) assay is no longer available in some settings. Here, we report the results of a clinical laboratory evaluation of alternative treponemal assays, including three *T. pallidum* hemagglutination assays (TPHAs), a *T. pallidum* IgG enzyme-linked immunosorbent assay (ELISA), and a detailed laboratory evaluation of the only TPHA with Australian regulatory approval. The clinical laboratory evaluation comprised 300 sera (120 TPPA reactive and 180 TPPA non-reactive) collected from individuals ≥18 years between 1 June 2021 and 1 June 2023. Median age was 33, 92% were from a sexually transmitted infection clinic, 71% were male, 15.3% were living with HIV and 5% were pregnant. Twenty-four percent had active untreated syphilis, 25% had prior treated syphilis, and 50.7% had no syphilis. Compared to TPPA, positive percent agreement (PPA) was 99.2% (95% confidence interval [CI], 95.4–99.9%), 100% (95% CI, 96.9–100%), 99.2% (95% CI, 95.4–99.9%), and 93.3% (95% CI, 87.4–96.6%), respectively, for the Arlington, Fortress, and Randox TPHAs, and Euroimmun IgG ELISA. Negative percent agreement (NPA) was 97.8% (95% CI, 94.4–99.1%) for each TPHA and 98.9% (95% CI, 96.0–99.8%) for the Euroimmun IgG ELISA. Clinical sensitivity of the Arlington and Fortress TPHAs for active untreated syphilis infection was equivalent to TPPA at 97.2% (95% CI, 90.4–99.5%) and clinical specificity of these assays was 99.3% (95% CI, 96.3–100%). In the evaluation of the NewBio TPHA, comprising 322 clinical serum and plasma samples and 22 quality assurance panel samples; overall PPA compared to TPPA was 100% (95% CI, 97.5–100%) and NPA 98.9% (95% CI, 94.1–98.9%). TPHAs are an acceptable alternative for confirmatory treponemal serology.

**IMPORTANCE:**

Rates of syphilis, including congenital syphilis, are increasing globally, resulting in substantial morbidity and neonatal mortality. A key pillar of syphilis control is timely and accurate diagnosis. Serology is the primary diagnostic test for syphilis. Serological testing for syphilis has been impacted by a withdrawal of the *Treponema pallidum* particle agglutination (TPPA) assay from several geographical regions, including Australia and Europe. Here, we describe the clinical and laboratory performance of alternative treponemal serological assays, with a focus on alternative agglutination assays (*Treponema pallidum* hemagglutination assays, TPHAs) as these assays comprise alternative antigens to commercial treponemal immunoassays, require a small volume of sample input and do not require specific instrumentation. This study demonstrates that TPHAs have excellent clinical and analytical performance characteristics and provides confidence that these assays are an acceptable alternative in settings that no longer have access to the TPPA.

## INTRODUCTION

Syphilis is a sexually transmitted infection (STI) caused by *Treponema pallidum* subsp. *pallidum* (1). In 2019, the estimated global prevalence of syphilis was 49.7 million, a 61% increase from 1990 (2). Rates of syphilis have risen significantly in many high- and middle-income countries over the past decade. In England, notifications more than doubled between 2013 and 2022 (3) (6.2–15.4 per 100,000), while they more than tripled in Australia and the United States (US) (7.6–24.3 per 100,000 and 5.5–17.7 per 100,000, respectively) (4, 5). Rates of congenital syphilis have also surged (4), with a 755% increase reported in the US between 2012 and 2021 (6). Syphilis can present with numerous symptoms (1) and lead to severe conditions including neurosyphilis (7). In pregnancy, syphilis infection can be transmitted vertically, causing neonatal morbidity and mortality (6).

Serological testing is the primary method for diagnosing syphilis (8), involving multiple tests to screen, confirm, and stage the infection. The reverse screening algorithm is commonly used in high-volume laboratories (8). This algorithm begins with a sensitive treponemal antibody immunoassay. If this assay is positive, it is followed by non-treponemal antibody tests. If the treponemal and non-treponemal results are discordant, a second confirmatory treponemal test is performed (9). In the United Kingdom and Australia, if the screening treponemal antibody result is positive, national guidelines recommend concurrent performance of non-treponemal and treponemal tests (10, 11). The *Treponema pallidum* particle agglutination (TPPA) assay is considered the preferred assay for confirmatory treponemal testing in international guidelines (10, 12). However, due to regulatory restrictions, this test has been withdrawn from some regions, including Europe and Australia (11). This has necessitated rapid updates to guidelines (11) and evaluations of alternative tests and diagnostic algorithms (13). Although the TPPA remains available in other regions including the US, there is currently only one TPPA manufacturer worldwide. As such, there are potential weaknesses in the supply chain for this important test.

A recent review suggested that the sensitivity and specificity of enzyme-linked immunosorbent assays (ELISAs) and chemiluminescent assays (CLIAs) may be comparable to agglutination assays (14). Updated national guidelines in regions where TPPA has been discontinued suggest that confirmatory testing may be performed with treponemal ELISA/CLIA (11). However, to maximize specificity, testing and confirmation with two treponemal assays with different antigens is recommended (11). There are many commercially available treponemal ELISA/CLIA, most using recombinant treponemal antigens (Tp15, Tp17, and Tp47) to detect IgM and/or IgG antibodies (14). However, since most of these assays share a common antigen (Tp17) (14), selecting a confirmatory test with distinct antigens can be difficult. Additionally, these assays often require significant specimen volumes, making them impractical as a third test in syphilis serology, particularly in pediatric testing, where sample volumes are often low. Further, many of these assays require expensive instruments, making them unfeasible for smaller clinical laboratories due to high initial costs and ongoing maintenance.

Accordingly, this study aimed to evaluate the performance of alternative syphilis confirmatory assays, including four *Treponema pallidum* hemagglutination tests (TPHAs) and one *T. pallidum* IgG ELISA, to address knowledge gaps in confirmatory testing pathways. Our work provides valuable data on the performance of syphilis serology assays, helping to improve testing strategies and address limitations in current diagnostic practices.

## MATERIALS AND METHODS

### Clinical validation

#### Study overview

**Fig 1:**
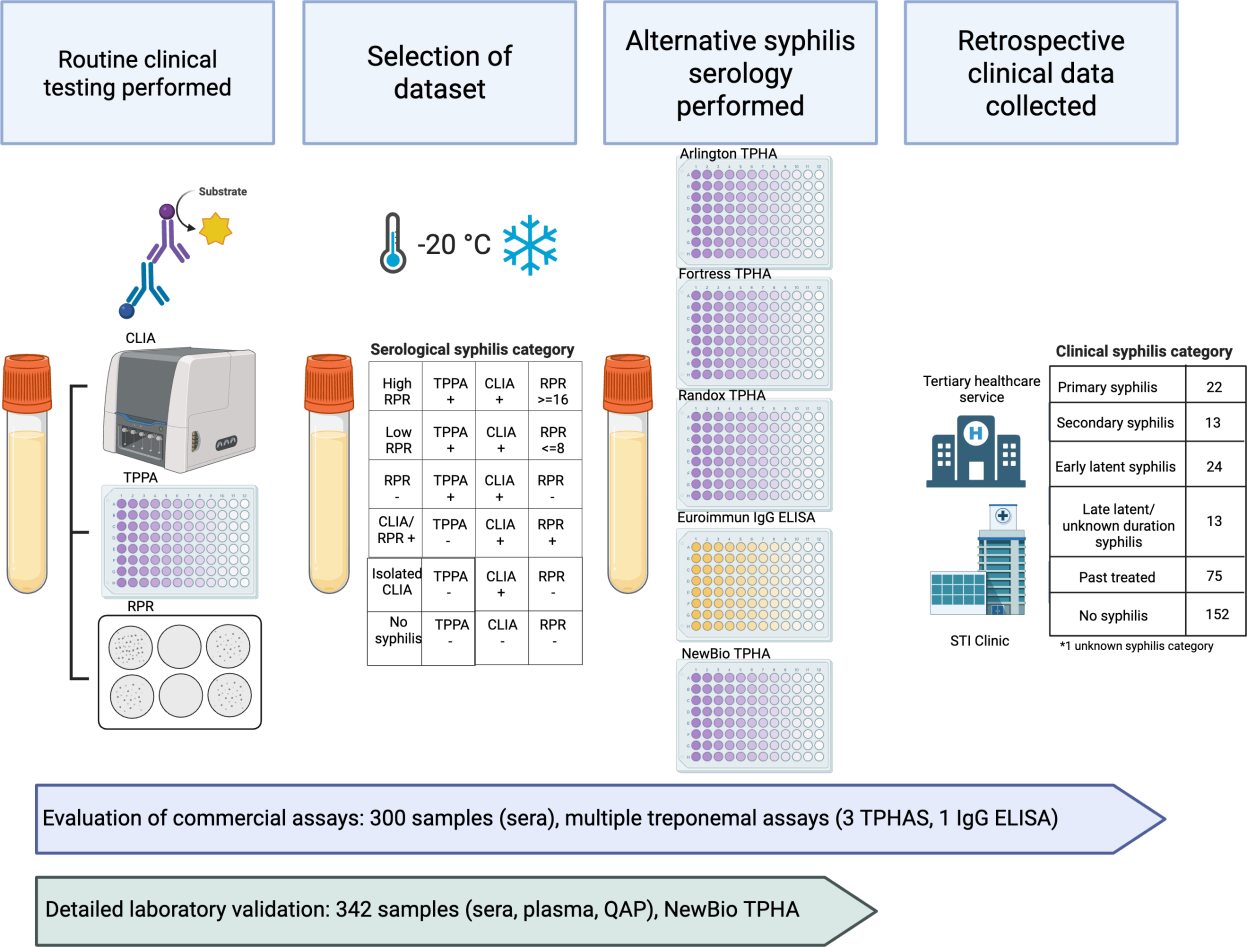
Visual representation of study methods. Part 1 comprised an evaluation of multiple commercially available alternative treponemal antibody assays using previously characterized clinical serum specimens. Part 2 comprised an evaluation of the only treponemal agglutination assay approved for use in our local setting using previously characterized clinical serum and plasma specimens and quality assurance program specimens. CLIA, chemiluminescent immunoassay; ELISA, enzyme-linked immunosorbent assay; IgG, immunoglobulin G; RPR, rapid plasma reagin; TPPA, *Treponema pallidum* particle agglutination. *Created in BioRender.* (15)

Our evaluation comprised two components described in Fig. 1; (i) an evaluation of commercially available alternative treponemal antibody assays; and (ii) an evaluation of the only treponemal agglutination assay approved for use in our local setting, comprising a panel of clinical serum and plasma samples, supplemented with quality assurance panel (QAP) samples, a cross-reactivity panel and assessment of assay precision.

#### Study sites and clinical samples

A retrospective laboratory evaluation was conducted at the Victorian Infectious Diseases Reference Laboratory (VIDRL) in Melbourne, Victoria, Australia, with clinical samples collected from two sites: Melbourne Sexual Health Centre (MSHC), the largest public STI clinic in Victoria, and Monash Health (MH), a tertiary-level healthcare service with over 2,000 inpatient beds in Melbourne including a large maternity care unit. VIDRL, the primary testing laboratory for multiple large sexual health clinics in Victoria, handles a high volume of syphilis serology. It also serves as the state reference laboratory for congenital syphilis and syphilis testing during pregnancy and performs confirmatory testing for referring laboratories.

The study population included adults ≥18 years of age from MSHC or MH who had syphilis serology performed at VIDRL between 1 January 2021 and 30 June 2023. Remnant sera from 300 clinical samples (120 TPPA reactive and 180 TPPA non-reactive) were included. Routine testing for syphilis at VIDRL comprises screening with a *T. pallidum* CLIA (Diasorin, Via Crescentio, Italy), Serodia TPPA (Fujirebio, Tokyo, Japan), and BD Macro-Vue rapid plasma reagin (RPR; Becton Dickinson, Franklin Lakes, USA). Local protocol states that, in addition to those samples with a reactive CLIA, TPPA, and RPR are also performed on samples when the *T. pallidum* CLIA signal/cut-off (s/co) ratio is 0.7–0.99, there is a past history of a reactive Diasorin CLIA or Serodia TPPA, a past history of syphilis or suggestion of early syphilis in the clinical notes, the sample has been referred for reference testing from another laboratory, or if specifically requested. As per local protocol, for samples that are CLIA reactive, TPPA non-reactive and RPR non-reactive without a prior history of reactive syphilis serology, repeat CLIA is performed in duplicate and repeat TPPA testing is performed in singlicate to confirm results. For CLIA reactive, TPPA non-reactive and RPR non-reactive cases with a prior history of a reactive CLIA and/or TPPA, repeat TPPA testing is performed in singlicate to confirm results. *T. pallidum* polymerase chain reaction (PCR) targeting the *polA* gene was performed as previously described (16) on swab samples from clinically relevant sites if indicated.

To ensure adequate performance of the assay in a wide range of clinically relevant stages of infection, the samples were obtained from the serological categories listed in Table S1. Associated data were collected, including gender, age, HIV status, antiretroviral status, HIV viral load, pregnancy status, gender of sexual partners in the past 12 months, *T. pallidum* PCR result performed in the 7 days prior to or following the day of serology sample collection, prior syphilis treatment and clinical stage of syphilis using criteria listed in Table S2. In brief, primary syphilis was defined as primary chancre plus one of (i) reactive syphilis serology on the day of specimen collection, (ii) reactive syphilis serology within 3 months after the day of specimen collection, or (iii) *T. pallidum* detected by PCR. Secondary syphilis was defined as systemic symptoms typical of syphilis with mucocutaneous lesions and reactive syphilis serology. Early latent syphilis was defined as asymptomatic with reactive syphilis serology and non-reactive syphilis serology results within the last 2 years. Late latent or unknown duration latent syphilis was defined as asymptomatic with reactive syphilis serology and no prior testing or prior testing 2 years or more since the last test. Past treated syphilis was defined as documented adequate treatment for syphilis in the patient chart, no signs of syphilis on the day of specimen collection and no subsequent diagnosis of syphilis in the 3 months after the day of specimen collection. No syphilis was defined as no diagnosis of syphilis on the day of testing (non-reactive syphilis serology and, if performed, *T. pallidum* not detected by PCR), no syphilis in the past medical history and no prior reactive syphilis serology. Based on the clinical stage of syphilis data, cases were classified into active untreated (primary, secondary, early latent and late latent or unknown duration latent syphilis clinical stages), prior treated syphilis (past treated syphilis clinical stage), and no syphilis (no syphilis clinical stage).

#### Serological testing

Remnant samples were retrieved from −20°C storage, thawed, tested using the alternative treponemal serological tests, and interpreted as per the manufacturer’s instructions for use (IFU) by two scientists trained in serological testing. Treponemal serology assays included three commercially available TPHA assays (i) Fortress Diagnostics TPHA Hemagglutination, Antrim, United Kingdom (UK); (ii) Randox Syphilis SYP-TPHA, Antrim, UK; (iii) Arlington Scientific TPHA Test, Springville, USA; and a commercial *T. pallidum* ELISA (Anti-*Treponema pallidum* ELISA IgG, Euroimmun, Lubeck, Germany) that utilizes a small sample input volume (10 µL). Key features of these assays are described in Table S3. Samples were tested using the Fortress, Randox, and Arlington TPHA and the Euroimmun Anti-*T*. *pallidum* IgG ELISA within one month of thawing from −20°C. Results were read and interpreted by two scientists. Samples in which there was a categorical disagreement in interpretation between scientists, results were discordant with the TPPA or results were equivocal (as defined by the manufacturer’s IFU) were resolved by repeat testing of the sample in duplicate, with the majority result from the three tests performed accepted as the result. Samples in which the control well was reactive were repeated in duplicate with the absorption procedure recommended by the manufacturer.

#### Data analysis

The primary outcome of the clinical validation study was the performance characteristics (positive percent agreement [PPA], negative percent agreement [NPA], positive predictive value [PPV], and negative predictive value [NPV]) of each alternative treponemal serological assay compared to the TPPA. Equivocal results were counted as positive for analysis. Any discrepancies between TPPA and the alternative treponemal serological assay were investigated further by reviewing the clinical syphilis stage, *T. pallidum* PCR and repeat serology results for the patient over a 6-month period to determine the true clinical status of the individual. Post hoc sensitivity analyses were also performed to assess the performance characteristics of each assay if equivocal results were excluded or counted as negative. In addition, predefined subgroup analyses were performed to determine the performance of these assays by (i) HIV infection status; (ii) pregnancy status, and (iii) clinical syphilis stage. Finally, sensitivity, specificity, NPV, and PPV were calculated for each alternative treponemal serological assay and the Serodia TPPA, compared to the overall clinical syphilis status of the individual (active untreated syphilis compared to no syphilis). Sensitivity of the alternative treponemal serological assays was also determined for the prior syphilis subgroup.

#### Detailed laboratory evaluation of the NewBio TPHA assay

Currently, the Newbio TPHA (Newmarket Biomedical, Kentford, UK) assay is the only commercially available assay with regulatory approval for use in clinical laboratories in Australia. Accordingly, to better inform national testing policies in Australia, we also undertook a detailed laboratory evaluation of this assay. Samples were tested as per the IFU for the Newbio TPHA assay (Table S3). Importantly, to comply with the IFU of this assay, all TPHA reactive or equivocal samples (as defined by the manufacturer’s IFU) were retested in duplicate, with the majority of the three results reported as the final interpretation. In addition, samples in which there was a categorical disagreement in interpretation between scientists or results were discordant with the TPPA were resolved by repeat testing of the sample in duplicate, with the majority result from the three tests performed accepted as the result. Samples in which the control well was reactive were repeated in duplicate with the absorption procedure recommended by the manufacturer. Equivocal samples were interpreted as having the presence of syphilis antibodies as per the Newbio TPHA IFU.

Samples from individuals who had syphilis serology performed at VIDRL between 1 January 2021 and 31 June 2024 were included in testing. Remnant samples that were collected as part of routine care were thawed from −20°C, stored at 2–8°C and tested within 1 month (Table S4). The data set described for the clinical validation was used for the TPPA reactive serum samples. A TPPA non-reactive panel was also tested, comprising 100 sera with *T. pallidum* CLIA, TPPA, and RPR performed as per laboratory procedures described above. As the NewBio TPHA assay is also validated for use on plasma samples, a plasma panel was also tested, which comprised 20 TPPA reactive and 20 TPPA non-reactive samples. In addition, samples from the WHO/CDC Quality Assurance Program (QAP) distributed between 1 January 2020 and 31 June 2024 were also tested, including 12 TPPA non-reactive and 10 TPPA reactive samples (Table S4).

Cross-reactivity assessment was performed to determine specificity. This panel comprised ten *T. pallidum* CLIA and RPR non-reactive samples that were reactive for HIV Ag/Ab, Hepatitis C antibody, Hepatitis B core antibody, Hepatitis B surface antibody, and Hepatitis A total antibody and 10 samples from pregnant individuals. Further, a reactive commercial quality control (QC) sample (MAS Syphilis Positive Control, Thermo Fisher Scientific, Waltham, USA) was repeated 10 times in a single run by a single operator to assess intra-assay variability. One reactive commercial QC sample (Thermo Fisher Scientific MAS Syphilis Positive Control) and one non-reactive QC sample (MAS Infectious Negative Control, Thermo Fisher Scientific, Waltham, USA) was repeated in triplicate over three runs over three different days by two different operators to assess inter-assay variability.

Performance characteristics (PPA, NPA, PPV, and NPV) of the NewBio TPHA were compared to the TPPA. Inter- and intra-run repeatability, inter-reader interpretation, and the repeatability of initially reactive and equivocal samples were also assessed.

### Statistical methods

Chi-squared and Fisher’s exact tests were used as appropriate to determine PPA, sensitivity, NPA, specificity, PPV, NPV, and 95% confidence intervals (CIs). Chi-squared, Fisher’s exact, and Mann-Whitney *U* tests were performed as appropriate to assess differences in demographic characteristics between the clinical cohorts (active untreated syphilis, prior syphilis, and no syphilis). Data were analyzed using Microsoft Excel Version 16.87 and Prism Version 10.2.0.

## RESULTS

### Characteristics of the data set

For the evaluation of commercially available alternative treponemal antibody assays, sera from 300 patients were included from individuals with a median age of 33 years (interquartile range 27–40); 213 (71.0%) were male, 46 (15.3%) were living with HIV, and 15 (5.0%) were pregnant. The majority of samples (92.3%) were from the STI clinic. Overall, 72 (24.0%) were clinically assessed as having active untreated syphilis infection, 75 (25.0%) had prior treated syphilis and 152 (50.7%) had no syphilis. Syphilis status was unknown for one sample. One hundred sixteen (38.7%) had been previously treated for syphilis, including 27 (9.0%) in the past 12 months (Table 1). The active untreated syphilis cohort was more likely to be male, people living with HIV (PLHIV) and gay and bisexual men who have sex with men than the no syphilis group (Table S5).

**TABLE 1:** Description of clinical validation data set^*b*^

Category	Variable	Number (*n* = 300)	Percentage
Demographics			
Age	Median [IQR]	33 [IQR 27–40]	N/A
Gender	Male	213	71.0%
	Female	81	27.0%
	Gender diverse	6	2.0%
HIV status	Positive	46	15.3%
	Negative	254	84.7%
Antiretroviral status (*n* = 46)	On ART	42/46	91.3%
	Not on ART	4/46	8.7%
HIV viral load	<20	28	60.9%
	20 to <200	9	19.6%
	200–1,000	1	2.2%
	>1,000	4	8.7%
	Unknown	4	8.7%
Pregnancy status	Pregnant	15	5.0%
	Not pregnant	285	95.0%
Sexual partners in past 12 months	Men who have sex with men	169	56.3%
	Men who have sex with men and women	13	4.3%
	Men who have sex with women	30	10.0%
	Women who have sex with men	63	21.0%
	Women who have sex with men and women	10	3.3%
	Women who have sex with women	4	1.3%
	Other^*a*^	11	3.7%
Clinical history			
Clinical setting	Sexual health clinic	277	92.3%
	Tertiary healthcare service	23	7.7%
Clinical syphilis stage	Primary	22	7.3%
	Secondary	13	4.3%
	Early latent	24	8.0%
	Late latent or unknown duration	13	4.3%
	Past treated	75	25.0%
	No syphilis	152	50.7%
	Unknown	1	0.3%
Syphilis treatment history	Treated in past 12 months	27	9.0%
	Treated >12 months prior	89	29.7%
	Never treated	183	61.0%
	Unknown	1	0.3%
Laboratory data			
TPPA reactive	High RPR	40	13.3%
	Low RPR	40	13.3%
	Negative RPR	40	13.3%
	Total	120	40.0%
TPPA non-reactive	CLIA and RPR reactive	1	0.3%
	CLIA reactive and RPR non-reactive	30	10.0%
	CLIA and RPR non-reactive	149	49.7%
	Total	180	60.0%
Indication for TPPA and RPR testing of CLIA non-reactive samples (*n* = 149)	CLIA s/co index ≥0.7	93/149	62.4%
	Prior elevated CLIA s/co index	5/149	34.2%
	Prior reactive TPPA or CLIA	22/149	14.8%
	Possible early syphilis in clinical notes	9/149	6.0%
	Past history syphilis in clinical notes	4/149	2.7%
	Referred from primary laboratory	3/149	2.0%
	Clinician request	5/149	3.4%
	Unknown	8/149	5.4%
*Treponema pallidum* PCR	Detected	21	7.0%
	Indeterminate	1	0.3%
	Not detected	26	8.7%
	Not performed	252	84.0%

^*a*^ Sexual partners of gender diverse individuals or sexual partners not described.

^*b*^ Abbreviations: ART, antiretroviral therapy; CLIA, chemiluminescent immunoassay; HIV, human immunodeficiency virus; IQR, interquartile range; N/A, not applicable; PCR, polymerase chain reaction; RPR, reactive plasma reagin; s/co signal-to-cutoff; TPPA, *Treponema pallidum* particle agglutination.

Overall, 120 (40.0%) samples were TPPA reactive and 180 (60.0%) were TPPA non-reactive (Table 1; Table S6). Of the 180 TPPA non-reactive samples, 2 (2 out of 180; 0.7%) were from individuals with *T. pallidum* PCR-positive primary syphilis collected on the same day as the syphilis serology sample. Twenty-five TPPA non-reactive samples (25 out of 180; 13.9%) were from individuals with a history of prior treated syphilis, including 13 (13 out of 25; 52.0%) with a documented prior history of a reactive TPPA at VIDRL, 3 (3 out of 25; 12.0%) with *T. pallidum* previously detected by PCR and 9 (9 out of 25; 36.0%) with a history of prior treated syphilis documented in the medical record.

Of the 149 *T. pallidum* CLIA non-reactive, TPPA and RPR non-reactive samples, 93 (93 out of 149; 62.4%) had CLIA, TPPA, and RPR testing performed due to an elevated CLIA s/co index (≥0.7) (Table 1). The median *T. pallidum* CLIA s/co index value for *T. pallidum* CLIA, TPPA, and RPR non-reactive samples was 0.7 (IQR: 0.43–0.76). Twenty-one of the 48 (16.0%) concurrently collected swabs for *T. pallidum* PCR testing (43.8%) were positive for *T. pallidum* DNA (Table 1).

### Performance characteristics of commercially available alternative treponemal assays

The PPA, NPA, PPV, and NPV for the assays included in the evaluation of commercially available alternative treponemal antibody assays compared to the Serodia TPPA assay are presented in Table 2; Table S7. Of the discordant results (TPPA non-reactive and TPHA reactive/equivocal), several were from individuals with prior treated syphilis, including three out of four from the Arlington TPHA, three out of four from the Fortress TPHA, and two out of four from the Randox. None of the two discordant Euroimmun IgG ELISA results (TPPA non-reactive and ELISA reactive) were from individuals with prior treated syphilis. Performance characteristics of the alternative treponemal serological assays compared to the TPPA among pregnant individuals and PLHIV are described in Table S8. No significant difference was detected in these subgroup analyses, noting that the number of pregnant individuals included in the study was limited.

**TABLE 2:** Performance characteristics of commercially available treponemal assays compared to Serodia TPPA assay and compared to syphilis status (active untreated syphilis, *n* = 72 vs no syphilis, *n* = 152)^*a*^^,^^*b*^

Assay	Performance characteristics of assay compared to Serodia TPPA assay				Performance characteristics of assay compared to clinical syphilis status			
	PPA (95% CI)	NPA (95% CI)	PPV (95% CI)	NPV (95% CI)	Sn (95% CI)	Sp (95% CI)	PPV (95% CI)	NPV (95% CI)
Serodia TPPA	N/A	N/A	N/A	N/A	97.2% (90.4–99.5)	100% (97.5–100)	100% (94.8–100)	98.7% (95.4–99.8)
Arlington TPHA	99.2% (95.4–100)	97.8% (94.4–99.1)	96.8% (91.9–98.7)	99.4% (96.9–100)	97.2% (90.4–99.5)	99.3% (96.3–100)	98.6% (92.4–99.9)	98.7% (95.4–99.8)
Fortress TPHA	100% (96.9–100)	97.8% (94.4–99.1)	96.8% (92.0–98.7)	100% (97.9–100)	97.2% (90.4–99.5)	99.3% (96.3–100)	98.6% (92.4–99.9)	98.7% (95.4–99.8)
Randox TPHA	99.2% (95.4–100)	97.8% (94.4–99.1)	96.8% (91.9–98.7)	99.4% (96.9–100)	95.8% (88.5–98.9)	98.7% (95.3–99.8)	97.2% (90.3–99.5)	98.0% (94.4–99.5)
Euro-immun IgG ELISA	93.3% (87.3–96.6)	98.9% (96.0–99.8)	98.3% (93.8–99.7)	95.7% (91.7–97.8)	93.1% (84.8–97.0)	98.7% (95.3–99.8)	97.1% (90.0–99.5)	96.8% (92.6–98.6)

^*a*^ *Note*: Equivocal results were considered positive for analysis.

^*b*^ Abbreviations: 95% CI, 95% confidence interval; ELISA, enzyme-linked immunosorbent assay; IgG, immunoglobulin G; N/A, not applicable; NPA, negative percent agreement; NPV, negative predictive value; PPA, positive percent agreement; PPV, positive predictive value; Sn, sensitivity; Sp, specificity; TPHA, *Treponema pallidum* hemagglutination assay; TPPA, *Treponema pallidum* particle agglutination assay.

Performance characteristics, including sensitivity, specificity, PPV, and NPV of the commercially available treponemal assays as well as the Serodia TPPA, were compared with clinical syphilis status (active untreated syphilis [*n* = 72] and no syphilis [*n* = 152] and are described in Table 2; Tables S5, S9 and S10. The sensitivity of the Arlington and Fortress TPHAs were equivalent to the TPPA. Sensitivity for each assay for each clinical stage of syphilis is also described in Table 3, with the Serodia TPPA and all TPHA assays having a sensitivity of 90.9% (95% CI, 72.2–98.4%) for primary syphilis, compared to 86.4% (66.7–95.3%) for the Euroimmun IgG ELISA. Persistently reactive treponemal serology among individuals with prior syphilis and the sensitivity of the commercial assays assessed for detection of prior syphilis is described in Table 3, with the Euroimmun IgG ELISA having the lowest number of persistently reactive results (54.7%) among this cohort and the Randox and Fortress TPHA having the highest number (68.0%). Key operational characteristics, including inter-reader variability, requirement for repeat testing, and number of equivocal and invalid results, are described in Table S11.

**TABLE 3:** Sensitivity of treponemal assays for detection of syphilis by stage (*n* = 72) and for those with prior syphilis (*n* = 75)^*a*^

Assay	Primary (*n* = 22) (95% CI)	Secondary (*n* = 13) (95% CI)	Early latent (*n* = 24) (95% CI)	Late latent/unknown (*n* = 13) (95% CI)	Prior syphilis (*n* = 75)
Serodia TPPA	90.9% (72.2–98.4)	100% (77.2–100)	100% (89.3–100)	100% (77.2–100)	66.7% (54.5–76.3)
Arlington TPHA	90.9% (72.2–98.4)	100% (77.2–100)	100% (89.3–100)	100% (77.2–100)	69.3% (58.2–78.6)
Fortress TPHA	90.9% (72.2–98.4)	100% (77.2–100)	100% (89.3–100)	100% (77.2–100)	70.7% (59.6–79.8)
Randox TPHA	90.9% (72.2–98.4)	100% (77.2–100)	100% (89.3–100)	92.3% (66.7–99.6)	69.3% (58.2–78.6)
Euroimmun IgG ELISA	86.4% (66.7–95.3)	92.3% (66.7–99.6)	100% (89.3–100)	92.3% (66.7–99.6)	60.0% (48.7–70.3)

^*a*^ Abbreviations: 95% CI, 95% confidence interval; ELISA, enzyme-linked immunosorbent assay; IgG, immunoglobulin G; TPHA, *Treponema pallidum* hemagglutination assay; TPPA, *Treponema pallidum* particle agglutination.

### Detailed laboratory evaluation of the NewBio TPHA

For the detailed laboratory evaluation of the NewBio TPHA, 342 samples were included. The clinical serum panel comprised 220 samples, including 120 (54.5%) TPPA reactive samples and 100 (45.5%) TPPA non-reactive samples. Of the 80 *T. pallidum* CLIA, TPPA, and RPR non-reactive samples, the median *T. pallidum* CLIA s/co index was 0.1 (IQR: 0.1–0.30). The clinical plasma panel comprised 20 TPPA reactive and non-reactive samples. The QAP included 10 TPPA reactive and 12 TPPA non-reactive samples, and the cross-reactivity panel comprised 50 TPPA non-reactive clinical sera with HIV antigen/antibody, Hepatitis A total antibody, Hepatitis B surface antibody, Hepatitis B core antibody or Hepatitis C antibody reactivity and 10 clinical sera from pregnant women.

### Performance characteristics of the NewBio TPHA

The PPA, NPA, PPV, and NPV of the NewBio TPHA compared to the Serodia TPPA assay for each panel included in the laboratory evaluation and pooled overall results are presented in Table 4. Of the three discordant results in the clinical serum panel (TPPA non-reactive and NewBio TPHA reactive), two out of three were from individuals with a documented history of a previously reactive TPPA. All 40 samples included in the plasma panel had concordant NewBio TPHA and TPPA results. One result was discordant (TPPA non-reactive and NewBio TPHA reactive) among the 22 sample QAP panel. This sample was also reactive when tested using the Arlington, Fortress, and Randox TPHAs. Of the 60 TPPA non-reactive samples included in the cross-reactivity panel, a single sample was discordant, testing equivocal on the NewBio TPHA assay. This sample was reactive for Hepatitis B core antibody.

**TABLE 4:** Performance characteristics of NewBio TPHA compared to TPPA in laboratory evaluation^*b*^

Result	TPPA reactive	TPPA Non-reactive	PPA (95% CI)	NPA (95% CI)	PPV (95% CI)	NPV (95% CI)
Serum clinical specimens (*n* = 219^*a*^)						
Reactive	118^*a*^	3	100% (96.9–100)	97.0 (91.6–99.2)	97.5% (93.0–99.3)	100% (96.2–100)
Equivocal	1	0				
Non-reactive	0	97				
Plasma clinical specimens (*n* = 40)						
Reactive	20	0	100% (83.9–100)	100% (83.9–100)	100% (83.9–100)	100% (83.9–100)
Equivocal	0	0				
Non-reactive	0	20				
Quality Assurance Program specimens (*n* = 22)						
Reactive	10	1	100% (72.3–100)	91.7% (64.6–99.6)	90.9% (62.3–99.5)	100% (74.1–100)
Equivocal	0	0				
Non-reactive	0	11				
Cross-reactivity specimens (*n* = 60)						
Reactive	0	0	N/A	98.3% (91.1–99.9)	N/A	100% (93.9–100)
Equivocal	0	1				
Non-reactive	0	59				
Overall results (*n* = 341^*a*^)						
Reactive	148^*a*^	4	100% (97.5–100)	97.4% (94.1–98.9)	96.8% (92.6–98.6)	100% (98.0–100)
Equivocal	1	1				
Non-reactive	0	187				

^*a*^ Single sample test result invalid due to persistent reactivity in control well after absorption procedure performed. Equivocal results were considered positive for analysis.

^*b*^ Abbreviations: 95% CI, 95% confidence interval; N/A, not applicable; NPA, negative percent agreement; NPV, negative predictive value; PPA, positive percent agreement; PPV, positive predictive value; Sn, sensitivity; Sp, specificity; TPHA, *Treponema pallidum* hemagglutination assay.

### NewBio TPHA reproducibility, repeatability, and inter-reader variability

Assessment of inter-assay and intra-assay variability was performed by testing of positive and negative QC samples in triplicate over three separate runs over three different days by two different operators to evaluate inter-assay variability, and 10 positive QC replicates in the same run, respectively. All positive and negative control results were reactive with a consistent level of reactivity recorded by both readers within and between each run, and no inter-reader variability was observed.

Repeat testing was performed on 159 samples (46.5%). Of the 132 samples that were reactive on initial NewBio TPHA testing, 131 (99.2%) were reactive on repeat testing in duplicate (Table S12). The single sample that was non-reactive upon repeat testing in duplicate was non-reactive on *T. pallidum* CLIA. A discordant result between *T. pallidum* CLIA and TPHA such as this would trigger repeat testing of the sample in duplicate to investigate the discordance according to local protocol. Of the 700 tests performed (including due to repeat testing in duplicate of positive, equivocal, invalid, and results discordant with the TPPA), inter-reader interpretation was concordant for 699 out of 700 (99.9%) tests (Table S12). The single discordant interpretation occurred for the invalid sample, which varied only in the level of reactivity graded for the control cells between readers.

## DISCUSSION

Here, we demonstrate that several commercial TPHAs have analytical performance characteristics comparable to those of Serodia TPPA. Importantly, when compared with active untreated syphilis status, three of the commercial TPHAs included in this study had equivalent sensitivity to the TPPA and two had a specificity of >99%. By contrast, the PPA of the *T. pallidum* IgG ELISA was <95% compared to the Serodia TPPA and had a clinical sensitivity <95%, including for diagnosis of primary, secondary, and late latent disease. The detailed laboratory validation of the single TPHA available in our setting demonstrated excellent PPA and comparable NPA compared to TPPA across a range of clinical sera, plasma, and quality assurance samples, including a specific cross-reactivity panel. It also performed well in the assessment of reproducibility, repeatability, and inter-reader variability, with only a small number of samples recording a final equivocal result.

Importantly, the majority of samples with an initial reactive result using the NewBio TPHA remained reactive on repeat testing. In our study, only a single sample was discordant with the initial reactive NewBio TPHA results on repeat testing. This sample was *T. pallidum* CLIA non-reactive (s/co < 0.10; TPPA non-reactive), with this discordance triggering repeat testing according to local testing protocols. These data suggest that local modification of the NewBio TPHA testing protocol from the IFU may be possible, with the omission of the requirement to perform repeat testing for reactive samples when using the assay for confirmatory testing of *T. pallidum* CLIA reactive samples. This protocol modification would substantially improve laboratory efficiency and result turnaround time.

The TPPA has been demonstrated to be highly sensitive and specific for *T. pallidum* infection (14). The sensitivity and specificity of the commercial TPHAs described in our study are similar to the performance characteristics of the TPPA described in a large laboratory evaluation by Park et al. that determined clinical syphilis status and compared the TPPA with five treponemal immunoassays and the fluorescent treponemal antibody (FTA-Ab) assay (17). This evaluation comprised samples from 262 individuals with active untreated syphilis, 294 samples from individuals with prior syphilis, and 403 from those without syphilis. The sensitivity and specificity of the TPPA were 95.4% and 100%, respectively, with the sensitivity and specificity of the alternative immunoassays reported between 96.9–98.5% and 82.6–98.5%, respectively. Although agglutination assays are manual tests, they are simple to perform, do not require specific instrumentation and can be used with a low sample input volume. They are also more specific and less subjective than alternative treponemal assays, such as the FTA-Ab assay (8).

There are limited studies of the clinical and laboratory performance of commercial TPHAs; however, our results are consistent with those observed in a study by Cole et al. (18). In this study, 235 treponemal positive samples, 250 negative blood donor samples, and 3 QC sera were included in an independent laboratory evaluation of four commercial TPHAs compared to the Serodia TPPA. The sensitivity of the commercial TPHAs ranged between 91.7% and 98.3% and specificity 99.6% and 100% in this study. The somewhat higher sensitivity of the TPHAs included in our study (95.8–97.2%) may be explained by inclusion only of individuals with active untreated syphilis in our analysis, compared to the inclusion of 121 positive specimens with unknown disease and treatment categories in the study by Cole et al. (18). The slightly lower specificity observed in our study is likely explained by the difference in the negative cohort included in these studies, with blood donors a much less challenging cohort than the STI clinic/tertiary health care/pregnancy samples included in our study. The negative samples included in our study comprised a large proportion of samples with a high CLIA s/co index consistent with the indication for TPPA and RPR testing of these samples. Importantly, the data set used in this study more closely mimics the sample types that undergo confirmatory treponemal testing when the reverse screening algorithm is used.

Other approaches to confirmatory treponemal antibody testing include the use of a secondary immunoassay. A recent study by Cheng et al. described the use of sequential treponemal immunoassays in blood donors (13). In this large study of 1,767,782 blood donor samples over a 2-year period, 1,850 samples were reactive on the Alinity s CMIA, with 1,456 of those non-reactive on the secondary treponemal assay, the Elecsys ECLIA. This was determined as a presumed Alinity false-positive rate of 0.08%; however, TPPA, RPR, and FTA-Ab testing were not performed on these samples, nor were clinical syphilis infection data or results of repeat testing reported for these individuals. Importantly, this change was implemented after an initial pilot where testing of 129 samples on the two different treponemal immunoassays was performed. In this smaller study, 105 samples had a discordant result (reactive Alinity s CMIA and non-reactive Elecsys ECLIA). Of these, 92.3% were determined to be false-positive Alinity results and 7.6% were determined to have likely syphilis infection based on reactive TPPA or FTA-Abs. Although this study demonstrates the feasibility of sequential treponemal immunoassay screening in the blood donor population, questions remain about the sensitivity and specificity of this approach for clinical diagnostic testing, particularly in populations with a high pre-test probability of syphilis infection, such as the STI clinic population.

There are several limitations of this study. First, testing was performed on samples that had undergone a single freeze-thaw cycle and were subsequently stored at 2–8°C for up to 1 month. These storage conditions are not recommended in the manufacturer’s IFU of the assay. The impact of these storage conditions on the results for samples that underwent a single-freeze thaw cycle and were stored at 2–8°C for 1 month is unclear. Other limitations include the retrospective nature of this study which may have introduced recall or reporting bias into the determination of clinical syphilis status, particularly with regard to prior syphilis status. In addition, information regarding clinical syphilis status was not available for the samples used in the NewBio TPHA evaluation. As such, clinical sensitivity and specificity could not be assessed. However, given the robust performance of this assay compared with the TPPA, it is expected that the clinical sensitivity and specificity would be similar to the other TPHAs included in this study. Further, a significant number of equivocal results were obtained for most of the assays included in this study. As per convention, we classified these results as “reactive” for the purposes of analysis of the performance of these assays; however, it was not clear from the IFU of a number of these assays how these equivocal results should be managed. As such, we performed post-hoc sensitivity analyses where equivocal results were either interpreted as negative or excluded from analysis. Overall, when considering the implementation of a new assay, it is preferable to use assays with fewer equivocal results as equivocal results may the complicate clinical interpretation of the test. Finally, it is important to note that determination of syphilis status requires incorporation of the patient’s clinical status (symptoms, risk factors, and prior history), molecular testing, and syphilis serology, as syphilis serology alone is an imperfect test (19–22).

This study demonstrates that TPHAs perform similarly to the TPPA and provides confidence that these assays are an acceptable alternative in settings that no longer have access to the TPPA. Importantly, three TPHAs had equivalent clinical sensitivity to the Serodia TPPA and two TPHAs had >99% clinical specificity. This study demonstrates the importance of including clinical data on syphilis status in the assessment of treponemal serology assays, where prior infection may result in persistent reactivity. This study also demonstrates the value of high-quality, independent laboratory evaluations of new tests performed by clinical laboratories when managing critical assay shortages or withdrawals of high-impact tests from the market. Recent global shortages in blood culture bottles and critical antibiotics, such as benzathine penicillin for the treatment of syphilis, have demonstrated the complexity faced by health systems when managing supply-chain and regulatory disruptions. Here, we have demonstrated that commercial TPHAs are an acceptable alternative to the gold-standard TPPA test used for confirmatory treponemal serology testing, and that a number of TPHAs have high clinical sensitivity and specificity for diagnosis of active untreated syphilis.

## SUPPLEMENTAL MATERIAL

Supplemental material jcm.01768-24-s0001.pdf Tables S1 to S12.

ASM does not own the copyrights to Supplemental Material that may be linked to, or accessed through, an article. The authors have granted ASM a non-exclusive, world-wide license to publish the Supplemental Material files. Please contact the corresponding author directly for reuse.
