# Supplementary material for: Alternative treponemal serology assays for diagnosis and confirmation of syphilis in a diagnostic laboratory: a retrospective evaluation of four agglutination assays and one ELISA
Source: J Clin Microbiol. 2025 May 9;63(6):e01768-24. doi: 10.1128/jcm.01768-24 (PMC12153291; doi:10.1128/jcm.01768-24)
Supplement: Supplemental material — Tables S1 to S12. [file jcm.01768-24-s0001.pdf]

SUPPLEMENTARY DATA

Table of Contents

Table S1: Syphilis serology categories included in clinical validation 2

Table S2: Criteria used to determine clinical syphilis stage 2

Table S3. Key operational considerations of treponemal serology assays included in clinical validation as per manufacturer's instructions for use 3

Table S4: Syphilis serology samples involved in the detailed laboratory validation of the NewBio TPHA 4

Table S5 Demographic characteristics of individuals by clinical syphilis status 4

Table S6. Results of alternative treponemal serology testing by TPPA result and clinical syphilis status. 5

Table S7. Performance characteristics of treponemal serology assays compared to Serodia TPPA (equivocal results considered as positive for analysis). 5

Table S8. Performance characteristics of treponemal serology assays compared to Serodia TPPA among HIV positive and pregnant individuals. 6

Table S9. Performance characteristics of treponemal serology assays for diagnosis of active untreated syphilis (n=72) and for those without syphilis (n=152) (equivocal results considered as positive for analysis). 6

Table S10. Sensitivity analysis of performance characteristics of treponemal serology assays compared to TPPA (considering equivocal results as negative for analysis, and excluding equivocal results from analysis, respectively) 7

Table S11. Operational considerations of analytical performance of treponemal serology assays included in clinical validation 7

Table S12. Operational considerations of analytical performance of NewBio TPHA in laboratory validation 7

Table S1: Syphilis serology categories included in clinical validation

CLIA result	TPPA result	RPR result	Category	Sample numbers
CLIA +	TPPA +	RPR ≥16	High RPR	40
CLIA +	TPPA +	RPR ≤8	Low RPR	40
CLIA +/-	TPPA +	RPR -	RPR negative syphilis	40
CLIA +	TPPA -	RPR -	Isolated positive CLIA	30
CLIA +	TPPA -	RPR +	CLIA/RPR positive	1
CLIA -	TPPA -	RPR -	No syphilis	149
Total				300

CLIA, chemiluminescent immunoassay; RPR, rapid plasma reagin; TPPA, *Treponema pallidum* particle agglutination.

Table S2: Criteria used to determine clinical syphilis stage

Syphilis category	Syphilis stage	Case definition
Active untreated	Primary syphilis	Primary chancre PLUS one of i) reactive syphilis serology on day of specimen collection, OR ii) reactive syphilis serology in the 3 months after the day of specimen collection, OR iii) *T. pallidum* detected by PCR
	Secondary syphilis	Systemic symptoms typical of syphilis PLUS mucocutaneous lesions AND reactive syphilis serology
	Early latent syphilis	Asymptomatic PLUS reactive syphilis serology AND non-reactive serology results within the last 2 years
	Late latent or unknown duration latent syphilis	Asymptomatic PLUS reactive syphilis serology AND no prior testing OR prior testing >2 years since the last test
Prior syphilis	Past treated syphilis	Documented adequate treatment for syphilis in the patient chart AND no signs of syphilis on the day of specimen collection AND no subsequent diagnosis of syphilis in the 3 months after the day of specimen collection.
No syphilis	No syphilis	No diagnosis of syphilis on the day of testing (non-reactive syphilis serology AND *T. pallidum* not detected by PCR if collected) AND no syphilis in the past medical history AND no reactive prior syphilis serology.

PCR: polymerase chain reaction. Reactive syphilis serology definition: reactive CLIA and TPPA +/- reactive RPR if no prior history of syphilis OR reactive CLIA and TPPA plus reactive RPR with a fourfold titre increase compared to previous sample if past treated syphilis.

Table S3. Key operational considerations of treponemal serology assays included in clinical validation as per manufacturer's instructions for use

Assay	Arlington TPHA	Fortress TPHA	Randox TPHA	NewBio TPHA	Euroimmun IgG ELISA
**Specimen**	Serum or plasma (EDTA). Serum preferred	Serum, plasma, CSF	Serum or plasma (EDTA)	Serum or plasma (EDTA)	Serum or plasma (EDTA, heparin, citrate)
**Volume**	10ul	10ul	10ul	10ul	10ul
**Storage**	If >5d, store at -20°C	24h at 4-8°C, long-term -20°C	24h at 4-8°C, 4w at -20°C	7d at 2-8°C, 1m at -20°C, up to 5 freeze-thaw cycles	14d at 2-8°C
**Preparation time**	30 minutes	30 minutes	30 minutes	30 minutes	2h
**Reading**	Visual reading after 45m incubation at room temperature	Visual reading after 45-60m incubation at room temperature or overnight	Visual reading after 45-60m incubation at room temperature or overnight	Visual reading after 45-60m incubation at room temperature	Colorimetric reading within 30m of stop solution
**Absorption procedure for reactive control well**	Yes	Yes	Yes	Yes	N/A
**Interpretation**	Serum must be used for repeat or confirmatory testing of reactive or equivocal plasma	Reactive samples should be retested in duplicate (2/3 reactive = positive)	Positive samples should be retested by quantitative test	Reactive samples should be retested in duplicate (2/3 reactive = positive)	Ratio <0.8 negative; Ratio ≥ 0.8 to <1.1 borderline; Ratio ≥1.1 positive
**Management of equivocal results**	No documented procedure	Repeatable equivocal samples should be considered positive	Retest	Repeatable equivocal samples should be considered positive	Recollect sample in 7d and test in parallel with initial sample

°C, degrees Celsius; CSF, cerebrospinal fluid; d, days; EDTA, ethyleneaminetetraacetic acid; ELISA, enzyme-linked immunosorbent assay; h, hours; IgG, immunoglobulin G; m, months; N/A, not applicable; TPHA, *Treponema pallidum* haemagglutination assay; ul, microlitres; w, weeks.

Table S4: Syphilis serology samples involved in the detailed laboratory validation of the NewBio TPHA

CLIA result	TPPA result	RPR result	Category	Sample numbers
**Clinical serum panel**				
CLIA +	TPPA +	RPR ≥16	High RPR	40
CLIA +	TPPA +	RPR ≤8	Low RPR	40
CLIA +	TPPA +	RPR -	RPR negative syphilis	40
CLIA +	TPPA -	RPR -	Isolated positive CLIA	20
CLIA -	TPPA -	RPR -	No syphilis	80
Total				220
**Clinical plasma panel**				
CLIA +	TPPA +	RPR ≥16	High RPR	1
CLIA +	TPPA +	RPR ≤8	Low RPR	3
CLIA +	TPPA +	RPR -	RPR negative syphilis	16
CLIA +	TPPA -	RPR -	Isolated positive CLIA	3
CLIA -	TPPA -	RPR -	No syphilis	17
Total				40
**Quality assurance panel***				
CLIA +	TPPA +	RPR ≥16	High RPR	5
CLIA +	TPPA +	RPR ≤8	Low RPR	5
CLIA -	TPPA -	RPR -	No syphilis	12
Total				22

CLIA, chemiluminescent immunoassay; RPR, rapid plasma reagin; TPPA, *Treponema pallidum* particle agglutination; +, positive; -, negative. *Quality assurance panel from WHO/CDC quality assurance program.

Table S5 Demographic characteristics of individuals by clinical syphilis status

Characteristics	Active untreated syphilis (n=72)	Prior syphilis only (n=75)	No syphilis (n=152)	P value*
Age (Median, IQR)	34 (29-39)	33 (28-42)	32 (26-39)	0.09
Male	60 (83.3%)	65 (86.7%)	88 (57.9%)	<0.001
Pregnant	6 (8.3%)	3 (4.0%)	5 (3.3%)	0.18
HIV positive	20 (27.8%)	14 (18.7%)	12 (7.9%)	<0.001
Gay and bisexual man who has sex with men	57 (79.2%)	64 (85.3%)	60 (39.5%)	<0.001

IQR, inter-quartile range. *Comparison of active untreated syphilis to no syphilis group

Table S6. Results of alternative treponemal serology testing by TPPA result and clinical syphilis status.

TPPA Result	Syphilis status	Arlington TPHA Result			Fortress TPHA Result			Randox TPHA Result			Euroimmun IgG ELISA Result		
		R	Eq	NR	R	Eq	NR	R	Eq	NR	R	Eq	NR
R n=120	Active untreated syphilis n=70	68	2	0	68	2	0	67	2	1	67	0	3
	Prior syphilis n=50	41	8	1	49	1	0	49	1	0	41	4	5
NR n=180	Active untreated syphilis n=2	0	0	2	0	0	2	0	0	2	0	0	2
	Prior syphilis n=25	1	2	22	2	1	22	2	0	23	0	0	25
	No-syphilis n=152	1*	0	150	1*	0	150	2	0	150	1	1	150
	Unknown n=1	0	0	1	0	0	1	0	0	1	0	0	1

ELISA, enzyme-linked immunosorbent assay; Eq, equivocal; IgG, immunoglobulin G; NR, non-reactive; TPHA, *Treponema pallidum* haemagglutination assay; R, reactive. *Single sample test result invalid due to persistent reactivity in control well after absorption procedure performed.

Table S7. Performance characteristics of treponemal serology assays compared to Serodia TPPA (equivocal results considered as positive for analysis).

Result	TPPA Reactive	TPPA Non-reactive	PPA (95% CI)	NPA (95% CI)	PPV (95% CI)	NPV (95% CI)
**Arlington TPHA**						
Reactive	109	2	99.2% (95.4-100)	97.8% (94.4-99.1)	96.8% (91.9-98.7)	99.4% (96.9-100)
Equivocal	10	2				
Non-reactive	1	175*				
**Fortress TPHA**						
Reactive	117	3	100% (96.9-100)	97.8% (94.4-99.1)	96.8% (92.0-98.7)	100% (97.9-100)
Equivocal	3	1				
Non-reactive	0	175*				
**Randox TPHA**						
Reactive	116	4	99.2% (95.4-100)	97.8% (94.4-99.1)	96.8% (91.9-98.7)	99.4% (96.9-100)
Equivocal	3	0				
Non-reactive	1	176				
**Euroimmun IgG ELISA**						
Reactive	108	1	93.3% (87.3-96.6)	98.9% (96.0-99.8)	98.3% (93.8-99.7)	95.7% (91.7-97.8)
Equivocal	4	1				
Non-reactive	8	178				

ELISA, enzyme-linked immunosorbent assay; IgG, immunoglobulin G; NPA, negative percent agreement; NPV, negative predictive value; PPA, positive percent agreement; PPV, positive predictive value; TPHA, *Treponema pallidum* haemagglutination assay; 95% CI, 95% confidence interval. *Single sample test result invalid due to persistent reactivity in control well after absorption procedure performed.

Table S8. Performance characteristics of treponemal serology assays compared to Serodia TPPA among HIV positive and pregnant individuals.

Assay	PPA (95% CI)	NPA (95% CI)	PPA (95% CI)	NPA (95% CI)
**Pregnancy status**	**Pregnant (n=15)**		**Not Pregnant (n=285)**	
Arlington TPHA	100% (70.1-100)	100% (61.0-100)	99.1% (95.1-100)	97.7% (94.2-99.1)
Fortress TPHA	100% (70.1-100)	100% (61.0-100)	100% (96.7-100)	97.7% (94.2-99.1)
Randox TPHA	88.9% (56.5-99.4)	100% (61.0-100)	100% (96.7-100)	97.7% (94.2-99.1)
Euroimmun IgG ELISA	88.9% (56.5-99.4)	100% (61.0-100)	93.7% (87.6-96.9)	98.9% (95.9-99.8)
**HIV status**	**HIV Positive (n=46)**		**HIV Negative (n=254)**	
Arlington TPHA	100% (87.9-100)	88.2% (65.7-97.9)	99.0% (94.7-100)	98.8% (95.6-99.8)
Fortress TPHA	100% (87.9-100)	83.3% (60.8-94.2)	96.7% (90.9-99.1)	99.4% (96.6-100)
Randox TPHA	100% (87.9-100)	83.3% (60.8-94.2)	98.9% (94.1-100)	99.4% (96.6-100)
Euroimmun IgG ELISA	100% (87.9-100)	94.4% (74.2-99.7)	91.3% (83.8-95.6)	99.4% (96.6-100)

ELISA, enzyme-linked immunosorbent assay; HIV, human immunodeficiency virus; IgG, immunoglobulin G; NPA, negative percent agreement; NPV, negative predictive value; PPA, positive percent agreement; PPV, positive predictive value; TPHA, *Treponema pallidum* haemagglutination assay; 95% CI, 95% confidence interval.

Table S9. Performance characteristics of treponemal serology assays for diagnosis of active untreated syphilis (n=72) and for those without syphilis (n=152) (equivocal results considered as positive for analysis).

Result	Current syphilis	No syphilis	Sensitivity (95% CI)	Specificity (95% CI)	PPV (95% CI)	NPV (95% CI)
**Serodia TPPA**						
Reactive	70	0	97.2% (90.4-99.5)	100% (97.5-100)	100% (94.8-100)	98.7% (95.4-99.8)
Equivocal	0	0				
Non-reactive	2	152				
**Arlington TPHA**						
Reactive	68	1	97.2% (90.4-99.5)	99.3% (96.3-100)	98.6% (92.4-99.9)	98.7% (95.4-99.8)
Equivocal	2	0				
Non-reactive	2	150*				
**Fortress TPHA**						
Reactive	68	1	97.2% (90.4-99.5)	99.3% (96.3-100)	98.6% (92.4-99.9)	98.7% (95.4-99.8)
Equivocal	2	0				
Non-reactive	2	150*				
**Randox TPHA**						
Reactive	67	2	95.8% (88.5-98.9)	98.7% (95.3-99.8)	97.2% (90.3-99.5)	98.0% (94.4-99.5)
Equivocal	2	0				
Non-reactive	3	150				
**Euroimmun IgG ELISA**						
Reactive	67	1	93.1% (84.8-97.0)	98.7% (95.3-99.8)	97.1% (90.0-99.5)	96.8% (92.6-98.6)
Equivocal	0	1				
Non-reactive	5	150				

ELISA, enzyme-linked immunosorbent assay; IgG, immunoglobulin G; NPV, negative predictive value; PPV, positive predictive value; TPHA, *Treponema pallidum* haemagglutination assay; TPPA, *Treponema pallidum* particle agglutination; 95% CI, 95% confidence interval. *Single sample test result invalid due to persistent reactivity in control well after absorption procedure performed.

Table S10. Sensitivity analysis of performance characteristics of treponemal serology assays compared to TPPA (considering equivocal results as negative for analysis, and excluding equivocal results from analysis, respectively)

Assay	Performance characteristics of assay considering equivocal results as negative				Performance characteristics of assay excluding equivocal results from analysis			
	PPA (95% CI)	NPA (95% CI)	PPV (95% CI)	NPV (95% CI)	PPA (95% CI)	NPA (95% CI)	PPV (95% CI)	NPV (95% CI)
Arlington TPHA	90.8% (84.3-94.8)	98.9% (96.0-99.8)	98.2% (93.7-99.7)	94.2% (89.8-96.7)	99.1% (95.0-100)	98.9% (96.0-99.8)	98.2% (93.7-99.7)	99.4% (96.9-100)
Fortress TPHA	97.5% (92.9-99.3)	98.3% (95.2-99.5)	97.5% (92.9-99.3)	98.3% (95.2-99.5)	100% (96.8-100)	98.3% (95.2-99.5)	97.5% (92.9-98.3)	99.4% (96.9-100)
Randox TPHA	96.7% (94.4-99.1)	97.8% (94.4-99.1)	96.7% (91.7-98.7)	97.8% (94.4-99.1)	99.2% (95.3-100)	97.8% (94.4-99.1)	96.7% (91.8-98.7)	99.4% (96.9-100)
Euro-immun IgG ELISA	90.0% (83.3-94.2)	99.4% (96.9-100)	99.1% (95.0-100)	93.7% (89.3-96.4)	93.1% (87.0-96.5)	99.4% (96.9-100)	99.1% (95.0-100)	95.7% (91.7-97.8)

ELISA, enzyme-linked immunosorbent assay; IgG, immunoglobulin G; NPA, negative percent agreement; NPV, negative predictive value; PPA, positive percent agreement; PPV, positive predictive value; TPHA, *Treponema pallidum* haemagglutination assay; 95% CI, 95% confidence interval.

Table S11. Operational considerations of analytical performance of treponemal serology assays included in clinical validation

Assay	Repeat testing required due to initial result (n=300)					Inter-reader varia-bility	Final Equivocal Result (n=300)	Final Invalid Result (n=300)
	Equiv-ocal	Invalid	TPPA discord-ance	Reactive	Total			
Arlington TPHA	20 (6.7%)	2 (0.7%)	5 (1.7%)	N/A	27 (9.0%)	22/342 (6.4%)	11 (3.7%)	1 (0.3%)
Fortress TPHA	17 (5.7%)	2 (0.7%)	5 (1.7%)	103	127 (42.3%)	12/348 (3.4%)	4 (1.3%)	1 (0.3%)
Randox TPHA	7 (2.3%)	2 (0.7%)	5 (1.7%)	N/A	14 (4.7%)	4/328 (1.2%)	3 (1.0%)	0 (0%)
Euro-immun IgG	6 (2.0%)	N/A	9 (3.0%)	N/A	15 (5.0%)	N/A	4 (1.3%)	0 (0%)

ELISA, enzyme-linked immunosorbent assay; IgG, immunoglobulin G; N/A, not applicable; TPHA, *Treponema pallidum* haemagglutination assay; TPPA, *Treponema pallidum* particle agglutination;

Table S12. Operational considerations of analytical performance of NewBio TPHA in laboratory validation

Repeat testing required due to initial result (n=342)					Inter-reader variability (n=700)	Final Equivocal Result (n=342)	Final Invalid Result (n=342)
Equivocal	Invalid	TPPA discordance	Reactive	Total			
22 (6.4%)	3 (0.9%)	2 (0.6%)	132 (38.6%)	159 (46.5%)	1 (0.1%)	2 (0.6%)	1 (0.3%)

TPPA, *Treponema pallidum* particle agglutination.
